# Structural and Functional Features of Developing Brain Capillaries, and Their Alteration in Schizophrenia

**DOI:** 10.3389/fncel.2020.595002

**Published:** 2021-01-15

**Authors:** Micaël Carrier, Jérémie Guilbert, Jean-Philippe Lévesque, Marie-Ève Tremblay, Michèle Desjardins

**Affiliations:** ^1^Axe Neurosciences, Centre de recherche du CHU de Québec – Université Laval, Québec, QC, Canada; ^2^Department of Molecular Medicine, Université Laval, Québec, QC, Canada; ^3^Axe Oncologie, Centre de recherche du CHU de Québec, Université Laval, Québec, QC, Canada; ^4^Department of Physics, Physical Engineering and Optics, Université Laval, Québec, QC, Canada; ^5^Division of Medical Sciences, University of Victoria, Victoria, BC, Canada; ^6^Department of Biochemistry and Molecular Biology, The University of British Columbia, Vancouver, BC, Canada; ^7^Neurology and Neurosurgery Department, McGill University, Montréal, QC, Canada

**Keywords:** schizophrenia, blood vessels, claudin-5, neurovascular unit, neurovascular coupling

## Abstract

Schizophrenia affects more than 1% of the world’s population and shows very high heterogeneity in the positive, negative, and cognitive symptoms experienced by patients. The pathogenic mechanisms underlying this neurodevelopmental disorder are largely unknown, although it is proposed to emerge from multiple genetic and environmental risk factors. In this work, we explore the potential alterations in the developing blood vessel network which could contribute to the development of schizophrenia. Specifically, we discuss how the vascular network evolves during early postnatal life and how genetic and environmental risk factors can lead to detrimental changes. Blood vessels, capillaries in particular, constitute a dynamic and complex infrastructure distributing oxygen and nutrients to the brain. During postnatal development, capillaries undergo many structural and anatomical changes in order to form a fully functional, mature vascular network. Advanced technologies like magnetic resonance imaging and near infrared spectroscopy are now enabling to study how the brain vasculature and its supporting features are established in humans from birth until adulthood. Furthermore, the contribution of the different neurovascular unit elements, including pericytes, endothelial cells, astrocytes and microglia, to proper brain function and behavior, can be dissected. This investigation conducted among different brain regions altered in schizophrenia, such as the prefrontal cortex, may provide further evidence that schizophrenia can be considered a neurovascular disorder.

## Introduction

Affecting 1% of the global population, schizophrenia (SCZ) is a disabling neurodevelopmental disorder that has seen little improvement in treatments over the last decades (Insel, 2010), leaving patients with a low quality of life (Ritsner et al., 2003). SCZ shows very high heterogeneity in the positive (i.e., hallucinations, delusions), negative and cognitive symptoms (i.e., incoherent alogia, affective flattening, anhedonia, learning, memory deficits) experienced by patients, which can be linked to dysfunction in different brain regions (Norris and Strickland, 2017; Glausier and Lewis, 2018). Many features of this disorder are being investigated and have been reviewed from different perspectives, such as the role of the immune system (Sekar et al., 2016; Hui et al., 2018), dopamine pathways (Weinstein et al., 2017), psychiatric deficits (Bora and Murray, 2014; Catalano et al., 2018) and sex differences (Bordeleau et al., 2019). Known risk factors include genetic variants (Marshall et al., 2017) and environmental factors (e.g., air pollution, stress, infection) (Huttunen and Niskanen, 1978; Gomes and Grace, 2017; Korpela et al., 2020). Another important aspect to consider for proper understanding of the pathogenesis of SCZ is the characterization of postnatal development of the brain and its vasculature, as proper establishment of the neurovasculature via bidirectional communication between endothelial cells (ECs) and central nervous system (CNS) cells (Segarra et al., 2018) is crucial for CNS development.

As the highway of the brain, the neurovasculature serves many roles for brain support by providing ions, oxygen, nutrients, and energy metabolites, while also allowing for communication between the periphery and the brain (McConnell et al., 2017). In homeostatic conditions, cerebral blood flow is regulated by the vasculature based on brain activity, increasing and reducing the flow in regions of high or low need (Peterson et al., 2011). To accomplish these functions, cerebral blood vessels need to develop and mature as an efficient network. Vascularization has been shown to be tightly guided by glial cells, such as microglia and astrocytes (Tata et al., 2015). Previous literature shows evidence of vascular impairments contributing to developmental disorders such as autism (Ouellette et al., 2020), and potentially SCZ (Najjar et al., 2017; Kealy et al., 2020). Although the role of these vascular alterations in SCZ is still not clear, one could hypothesize that vascular changes during development affect the establishment of the blood vessel network, leading brain maturation down a path that eventually results in the symptoms experienced by SCZ patients. This review will underline the current view on the vascular hypothesis through discussing normal postnatal development of the neurovascular unit (NVU) in humans and animal models, the establishment of the neurovascular coupling, as well as the misshaping of this development as a potential contributor to SCZ pathogenesis.

## Development of the Neurovascular Unit

The NVU is a relatively recent concept (Iadecola, 2017) that refers to the cellular components [e.g., endothelial cells (ECs), pericyte and astrocyte] that contribute to the functional relationship between brain cells and cerebral vasculature (Coelho-Santos and Shih, 2020) with each cell type having their specific molecular signature (Vanlandewijck et al., 2018). This relationship notably allows for neurovascular coupling (NVC) between neuronal activity and blood flow and the establishment of a properly selective blood-brain barrier (BBB) required to protect the brain against homeostatic disturbance from the periphery (Bell et al., 2019; Sweeney et al., 2019).

### Neurovascular Coupling During Normal Development

Although still an area of active research, the various cellular elements of the BBB play a role in coupling neuronal activity to vascular tone and cerebral blood flow. Astrocytes can react to glutamatergic synaptic signaling by producing vasoactive compounds that cause pericytes to dilate capillaries (Hall et al., 2014; Mishra et al., 2016; Kisler et al., 2017). Capillary ECs can also detect potassium ionic currents and subsequently propagate a vasodilatory signal to upstream arterioles (Longden et al., 2017). Various neuronal subtypes directly signal to the vasculature by producing vasodilative or vasoconstrictive molecules (Uhlirova et al., 2016), for example nitric oxide release by glutamatergic neurons was proposed to suppress release of the vasoconstrictor 20-hydroxyeicosatetraenoic acid by astrocytes (Hall et al., 2014). This neurovascular coupling (NVC) explains the relationship between neuronal activity and the tight modulation of local oxygen/glucose concentration (Iadecola, 2017) and can provide an indirect measure of metabolic demand, which is altered in certain disorders including SCZ (Zhu et al., 2017).

NVC is also the basis for hemodynamic based non-invasive imaging of brain activity. When neuronal activity elicits an increase in blood flow in a given brain region, the rate of oxygen delivery exceeds the rate at which it is consumed, leading to a localized increase in oxyhemoglobin concentration concomitant with a decrease in deoxyhemoglobin (HbR) concentration (Buxton, 2013). With hemodynamic based functional imaging techniques, this change in oxygenation can be measured and used as a proxy for neuronal (and glial) activity. Among those techniques, functional magnetic resonance imaging (fMRI) and near infrared spectroscopy (NIRS) are the most commonly used for imaging neurovascular development in infants (Kozberg and Hillman, 2016; Hendrikx et al., 2019). In fMRI, changes in HbR concentration create the positive (HbR decrease) or negative (HbR increase) blood oxygenation level-dependent (BOLD) signal (Ogawa et al., 1992; Kim and Ogawa, 2012). Optical functional techniques, such as NIRS and its more invasive equivalent used in rodents, intrinsic optical signals (IOS), also measure HbR as well as oxy- and total hemoglobin concentration changes. Performing MRI in infants is still very challenging because of its sensitivity to motion artifacts (Dean et al., 2014), whereas NIRS offers a portable alternative for measuring functional hemodynamic signals in the cortex at low cost and which can be used in multiple experimental environments, even in schools (Soltanlou et al., 2018; Whiteman et al., 2018).

MRI and NIRS have shown great potential to measure hemodynamic signals longitudinally (Demirci et al., 2008; Yang et al., 2019) with growing literature investigating development as gathered in Table 1. This table compares results from previous studies in which task-evoked hemodynamic responses were measured in healthy young children or rodents using fMRI or NIRS/IOS.

**TABLE 1:** Summary of 20 years of studies investigating hemodynamic responses at several stages of homeostatic cerebrovascular development.

**fMRI studies**								
**References**	**Species**	**State**	**Stimulation**	**Age**				**BOLD results**
Born et al. (2000)	Human	Asleep/Awake	Visual	48 weeks				↑
				56 weeks				↓
Yamada et al. (2000)	Human	**–**	Visual	0–7 weeks				↑
				8–22 weeks				↓
Anderson et al. (2001)	Human	Awake	Auditory	40–50 weeks				↑
				50 weeks				↓
Sie et al. (2001)	Human	Sedated	Visual	18 months				↓
Born et al. (2002)	Human	Sedated	Visual	4–71 weeks				↓
Erberich et al. (2006)	Human	Sedated	Somatosensory	28–46 weeks				↓
Colonnese et al. (2008)	Rats	Sedated	Somatosensory	P13 to adulthood				↑
Heep et al. (2009)	Human	Sedated	Somatosensory	Preterm infant (26.5 weeks)				↓
				Term infant (39 weeks)				↓
Arichi et al. (2010, 2012)	Human	Sedated	Somatosensory	Preterm Term				↑ ↑

**Optical imaging studies**								
**References**	**Species**	**State**	**Stimulation**	**Age**	**HbO**	**HbR**	**HbT**	**BOLD equivalence**

Sakatani et al. (1999)	Human	Awake	Visual	3 years	**–**	**–**	**↑**	**–**
Hoshi et al. (2000)	Human	Asleep	Visual	4–5 days	**↑**	**↑**	**↑**	–
						None		
						↓		
Zaramella et al. (2001)	Human	Awake/Asleep	Auditory	0–7 weeks	–	–	**↑**	–
Taga et al. (2003)	Human	Awake	Visual	2–4 months	**↑**	↓	**–**	**↑**
Kusaka et al. (2004)	Human	**–**	Visual	4–16 weeks	↓	**↑**	**↑**	↓
Watanabe et al. (2008)	Human	Awake	Visual	2–4 months	**↑**	↓	**–**	**↑**
Karen et al. (2008)	Human	Asleep	Visual	2–9 days	**↑**	↓	**↑**	**↑**
Liao et al. (2010)	Human	Asleep	Visual	2 days	**↑**	↓	**↑**	**↑**
Kozberg et al. (2013)	Rats	Anesthetized	Somatosensory	P12–P13	↑	**↓**	↑	↑
				(∼1 year human in humans)	↓	↑	↓	↓
Sintsov et al. (2017)	Rats	Non-sedated	Somatosensory	0–3 months (∼8 years in humans)	**↑**	↓	**–**	**↑**

The up or down arrows indicate an increase or a decrease, respectively, in the value of the measure of blood oxygen level dependent (BOLD) signal, oxyhemoglobin (HbO), deoxyhemoglobin (HbR) and total hemoglobin (HbT) during the activation period in comparison to the resting period. Multiple arrows in the same box signify different responses observed within the group of the study and no change between those two states is identified by “None.” Parameters not reported in these studies are identified with a hyphen (-). Equivalence between rat and human ages were estimated based on (Sengupta, 2013).

Overall, these results are difficult to properly interpret. Although it is known that the hemodynamic response is necessary to induce vessel remodeling (Lucitti et al., 2007), the timeline of developmental patterns of the various components of NVC are not all well-defined, making it difficult to know if the varied hemodynamic responses observed are caused by altered neuronal activity in infants or an immature NVU. Second, as was previously noted (Harris et al., 2011), the lack of standardization in imaging parameters and stimulation paradigms adds many confounding variables when looking for consistent trends in results from functional imaging studies. Given the vascular component of SCZ, it can be investigated using techniques reported in Table 1. In our review of the literature on the hemodynamic response in SCZ patients investigated using NIRS (Ikezawa et al., 2009; Takizawa et al., 2009; Fujita et al., 2011; Kinou et al., 2013; Pu et al., 2015, 2016; Noda et al., 2017) and fMRI (Barch et al., 2003; Kircher et al., 2004; Tregellas et al., 2004, 2009; Ford et al., 2005; Dyckman et al., 2011; Mayer et al., 2013, 2016; Hanlon et al., 2016), no studies were found during development, a question that should be addressed to better understand NVC deficits in SCZ. The structure of the NVU is also a growing field for SCZ research (Villabona-Rueda et al., 2019).

### Development of the Capillary Network

During postnatal development, bidirectional communication between brain cells and the nascent vasculature ensures that capillaries grow side-by-side with the maturing neurons and glial cells so that the latter are provided with sufficient energy substrates (Paredes et al., 2018). This results in a dense mesh of capillaries matching the metabolic demand of the neurons and glial cells they support (Craigie, 1945; Weber et al., 2008; Lacoste et al., 2014). In rodents, at birth, the capillary bed is sparse, but goes through a rapid expansion in the first few postnatal weeks. Studies examining capillary growth from birth to adolescence in rodents have consistently shown more than a twofold postnatal increase in measures such as vessel density and volume compared to neurons density and branching before the growth stabilizes at postnatal day (P)20 (Keep and Jones, 1990; Wang et al., 1992; Zeller et al., 1996; Harb et al., 2013). A similar increase is seen in postnatal primates, in which relative vascular volume can double between birth and adulthood, reducing the distance between tissue and the vasculature by 32%. This doubling occurs mostly via angiogenesis and partly from the lengthening of existing vessels (Risser et al., 2009). This vascular increase is thought to originate almost solely from the capillary bed, as the network of larger penetrating arterioles and ascending venules is stable throughout postnatal development (Norman and O’Kusky, 1986; Risser et al., 2009). Interestingly, an earlier study in young rats showed that the vascularization of the capillary bed is not a continuous process, but rather occurs in distinct bouts of intense sprouting between P0 and P4, P7 and P8, at P10 and at P14, across the cerebral cortex (Rowan and Maxwell, 1981) but not the cerebellum (Craigie, 1924). The temporal pattern of sprouting was different across cortical layers, but always more intense in the middle layers, peaking within cortical layer 4 at adulthood (Harrison et al., 2002; Blinder et al., 2013).

Angiogenesis in the capillary bed is highly adaptive during early development. In rodents, enhanced sensory stimulation of the whiskers or complex experiences (e.g., vision) in the first postnatal month can increase capillary density in the somatosensory and visual cortices, respectively (Black et al., 1987; Lacoste et al., 2014). On the other hand, both sensory deprivation and hyperstimulation during that period can result in lower capillary density (Lacoste et al., 2014; Whiteus et al., 2014) without measurable changes in neuronal density in the regions analyzed. The pial vasculature for its part does not seem to adapt to sensory stimuli (Adams et al., 2018).

Following an early critical window, the microvasculature becomes less adaptive: for example, Whiteus et al. (2014) showed that the decreases in capillary density observed following chronic hyperstimulation by repetitive sounds, whisker deflection or motor activity in mice neonates (P15) can be restored if the perturbations were stopped after 5 days, but not if they were sustained for 15 days. Chronic hypoxia, which can induce robust angiogenesis in young mice during the second week of life, has also been shown to stop evoking capillary responses in the somatosensory and motor cortices after 3 months of age (Harb et al., 2013).

### Development of the Cellular Components of the NVU

**FIGURE 1:**
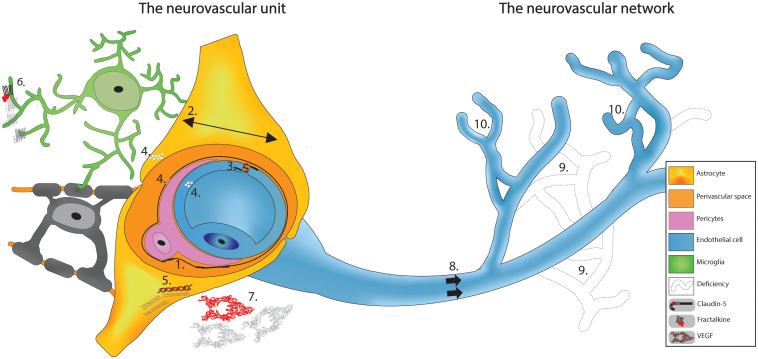
Schematic based on cumulative observations made on SCZ patients in the PFC region. The vasculature appears to have deficits at the neurovascular unit where we see: (1) Thickening and deformation of basal lamina. (2) Increase in the area of the astrocytic endfeet. (3) Haplodeficiency of claudin-5. (4) Cytoplasm vacuolation; and (5) Reduced GFAP labeling. When investigating the vasculature as a network surrounded by glial cells, cumulative evidences shows: (6) Diminished fractalkine signaling. (7) Decreased VEGF expression. (8) Reduced cerebral blood flow. (9) Lower capillaries density; and (10) Abnormal arborization. Altogether, these findings propose a potential vascular signature for SCZ that might explain the neuronal (and glial) functional deficits.

The main components of the NVU (Figure 1), ECs, exert functions such as the active transport of ions and nutrients through the BBB via membrane transporters whose levels vary during development. Expression of the P-glycoprotein (PGP) efflux transporter, which is hardly detectable at birth, is upregulated throughout the first postnatal month in mice (Daneman et al., 2010). ECs also upregulate the glucose transporter (GLUT) 1 in the second week to reach adult levels by P30 in rats (Harik et al., 1993; Vannucci and Vannucci, 2000).

The second main component of the NVU is astrocytes and their endfeet. In rodents, astrocytes start to be present in the cortex shortly after birth, and their endfeet typically fully ensheath capillaries by P15 (Mathiisen et al., 2010; Gilbert et al., 2019). In parallel, the gliovascular interface undergoes maturation, as protein complexes at the junction between perivascular astrocytic endfeet are assembled between P10 and P15 (Gilbert et al., 2019). The timing of astrocyte appearance in the cortex differs between species. In humans, this begins embryonically (El-Khoury et al., 2006). When astrocytes appear postnatally, the BBB is already functional meaning that astrocytes are not required for BBB function but rather seem to have a role in BBB maintenance later in life (Daneman et al., 2010). In addition, microglia were shown to ensheath the basement membrane of capillaries and contribute to the glia limitans, although their roles in the BBB formation and maintenance remain largely elusive (Bisht et al., 2016; Joost et al., 2019).

In contrast, pericytes coverage of capillaries is already established in neonatal rodents and is vital for BBB establishment, playing a role in proper tight junction orientation (Daneman et al., 2010; Ben-Zvi et al., 2014). Furthermore, during postnatal angiogenesis, pericytes are recruited to induce the formation of new capillaries via platelet-derived growth factor signaling in mice (Lindblom et al., 2003). Pericyte proliferation decreases steadily in mice from birth to P25 in the somatosensory and motor cortex (Harb et al., 2013). Initially, ECs express cluster of differentiation 146 (CD146) in order to upregulate claudin-5 forming the BBB. Expression of CD146 by pericytes promote their migration toward the ECs which in turn release transforming growth factor beta 1, down-regulating endothelial CD146 to reduce the expression of claudin-5 (Chen et al., 2017). Of the many components required for the development of the NVU, claudin-5, the dominant component of tight junctions forming the BBB, is already expressed in capillary ECs at P0 (Ek et al., 2006; Greene et al., 2019). In mice, its production increases more than threefold by P15 before stabilizing, indicating continued postnatal maturation of the BBB (Gilbert et al., 2019). Claudin-5 deficiency, resulting in BBB dysfunction, is causal in animal models of stress and depression (Menard et al., 2017; Pearson-Leary et al., 2017). Furthermore, mutation in claudin-5 is also seen in SCZ human patients (Omidinia et al., 2014) with dysfunction linked to change in other tight junction proteins such as ZO-1 and occludin (Maes et al., 2019; Greene et al., 2020).

### Cellular, Vascular, and Genetic Dysfunction in SCZ

SCZ is recognized to be linked to genetic vulnerabilities (Strawbridge et al., 2018; Chen et al., 2019; D’Ambrosio et al., 2019) (also reviewed in Comer et al., 2020a) and environmental factors during adolescence and into young adulthood (Pulver, 2000; Gomes and Grace, 2017; Qiu et al., 2019; Barichello et al., 2020). On the vascular level, genetic mutation on the chromosome 22q11 results in the loss of about 40 genes, one gene of interest being claudin-5 (Graw et al., 2012; Tang et al., 2014; Thompson et al., 2017). In mice engineered with a mutation in 22q11, claudin-5 expression is reduced by 75% in ECs, which was reproduced in cell culture (Greene et al., 2018). Furthermore, using MRI in SCZ patients, the 22q11 mutation was associated with decreased brain volumes for both total grey (*g* = −0.81) and total white matter (*g* = −0.81) calculated by a meta-analysis of between-group differences in mean volumes, representing the effect size (*g*) (Rogdaki et al., 2020). Considering that most investigations on vascular alterations in patients with SCZ are done using *post-mortem* tissue (McGlashan, 2011; Harris et al., 2012; Katsel et al., 2017), it is difficult to have a good idea on the temporal development of those deficits. To our knowledge, no longitudinal studies have been performed on the vascular aspect of SCZ, a question that remains to be addressed in the field. When the NVU and the BBB are altered in SCZ, then the vasculature would be unable to answer neuronal and glial cells engaging in their normal activities. A *post-mortem* study showed cardiovascular disorders as the primary cause of death in SCZ patients (Sweeting et al., 2013). More clinical evidence was extensively reviewed by Najjar et al. (2017). Notably, patients show elevation in CSF albumin (higher ratio of CSF-albumin to P-albumin), IgG, IgM, S100B and in several vascular endothelial adhesion molecules (soluble platelet selectin, serum L-selectin, integrin αIIbβIIIa, receptors on platelets) as well as decreases in vascular endothelial growth factor (VEGF) (Najjar et al., 2017; Melkersson and Bensing, 2018). In living human studies using dynamic contrast-enhanced (DCE)-MRI to study BBB integrity of the hippocampus, investigations pertaining to dementia and related disorders are extensive (Raja et al., 2018; Nation et al., 2019) but have not yet been targeted at the specific case of SCZ.

### Vascular Dysfunction in SCZ PFC

Brain imaging in SCZ patients investigating the hemodynamic response has been performed using fMRI (Hanlon et al., 2016). Although data is lacking about the prodromal stage, many vascular correlates of the disease have been identified. The PFC has been the subject of a great number of studies detailing the vasculature in SCZ, but is not the only region implicated. Whole brain analysis using inflow-based vascular-space-occupancy MRI also show significant reduction in arterial cerebral brain volume in temporal cortex grey matter of SCZ patients (Hua et al., 2017). Studies using different MRI sequences found reduced CBF in the frontal lobe (Malaspina et al., 2004), temporal lobe (Kindler et al., 2015), parietal lobe (Scheef et al., 2010) and occipital lobe (Pinkham et al., 2011).

SCZ patients also show morphological and functional alteration in glial cells present in this region, such as microglia (Bordeleau et al., 2019) and astrocytes (Abbink et al., 2019). Dark microglia, classified as such by their electron dense cytoplasm, have been found in numerous pathological conditions including in patients with SCZ and animal models of schizophrenia-like disorder simulated with the viral mimic poly I:C (Hui et al., 2018; St-Pierre et al., 2020). These altered microglia make extensive interactions with the NVU and have been suggested to take over astrocytic functions in SCZ (St-Pierre et al., 2020). Investigations of astrocytes in SCZ patients revealed larger astrocytic endfeet covering vessels (Uranova et al., 2010). This could be a compensation mechanism for the decreased astrocytic density seen in SCZ patients (Najjar et al., 2017), resulting in missing NVU components (Figure 1). There are also myelination deficits in patients with SCZ, implicating another glial cell type, oligodendrocytes (Raabe et al., 2018). A recent review has highlighted the need for NVU integrity to promote oligodendrocyte survival, potentially explaining the myelination deficit in SCZ (Hamanaka et al., 2018).

All three glial cell types appear to be key players in SCZ as covered in reviews focused on the subject (Bernstein et al., 2015). Astrocytes and microglia play key roles in controlling cerebral blood flow in a calcium dependent way as shown in mice (Mulligan and MacVicar, 2004; Mishra et al., 2016; Kleinberger et al., 2017). Overall, defects in the PFC vasculature and alterations in glial cells in SCZ investigations keep emerging, allowing us to both revisit existing and draw new hypotheses on its pathophysiology.

## Discussion

### The Vascular Hypothesis

Although many of the findings discussed above are recent, the vasculature hypothesis of SCZ is not. As highlighted in a brief history (Meier et al., 2013) based on a century old hypothesis (McGlashan, 2011), symptoms of SCZ could possibly be explained by cerebral microvasculature damages (Hanson and Gottesman, 2005). A possible mechanism is systemic inflammation shown in SCZ patients (Cai et al., 2020) coming from environmental factors (e.g., pollution, stress, nutrition induced gut-brain axis dysbiosis, viral infection, maternal immune activation) and genetic predisposition as the source of perturbation (Comer et al., 2020a). This inflammation is detrimental to the development of the vasculature, possibly already weakened by genetic mutation resulting in cellular damage (Hanson and Gottesman, 2005). The affected cells of the NVC would fail to maintain BBB integrity resulting in leakiness, associated with homeostatic disturbance from the periphery (e.g., inflammatory mediators and cells), and blood flow reduction providing limited oxygen and nutrient supply to the brain, impairing brain maturation. This mechanism is consistent with evidence seen in other disorders such as Alzheimer (Korte et al., 2020) and could explain the higher probability of neurodegenerative disorder in diabetic patients in which many vascular anomalies are observed (Nelson et al., 2016). Alterations in glial cells (mainly microglia and astrocytes) could contribute to this neurovascular fragility (Figure 1). Growing evidence place the PFC as central in this hypothesis because multiple investigations on SCZ patients found vascular defects in this particular region, ranging from decreases in claudin-5 (Greene et al., 2018), reductions in VEGF signaling (Fulzele and Pillai, 2009; Huang et al., 2020), a less dense capillary network (Uranova et al., 2013), to oversimplified angioarchitecture (Senitz and Winkelmann, 1991; Uranova et al., 2010), and other ultrastructural defects (Figure 1; Webster et al., 2001; Uranova et al., 2010; Ishizuka et al., 2017; Hill et al., 2020). As many key components of NVC are impacted by SCZ, it is not surprising that one of the most consistently observed neurovascular correlates of the illness is hypo-activity in PFC regions and in the left superior temporal gyrus, as revealed by a recent systematic review of both task and resting-state fMRI cross-sectional studies in first-episode SCZ patients (Mwansisya et al., 2017).

Although this hypothesis places the vasculature as a central element of SCZ, it is not clear whether the structural and functional abnormalities in blood vessels are a cause or a consequence of the cortical maturation deficiency. Growing evidence shows that an abnormal pruning of synapses and neurons by microglia potentially causes the cortical deficiency associated with SCZ (Sellgren et al., 2019). This altered removal of synapses is still partially unexplained, although it may result from dysfunctional fractalkine, triggering receptor expressed on myeloid cells 2 or complement signaling (Paolicelli et al., 2011; Hoshiko et al., 2012; Schafer et al., 2012; Filipello et al., 2018), all involved in microglia-mediated synaptic pruning. Complement is a prime suspect as work has shown upregulation of complement 4 protein in SCZ patients’ brain (Sekar et al., 2016) and mouse models of SCZ (Comer et al., 2020b). When compared to other neurodegenerative disorders, the SCZ vascular hypothesis has similitudes with the recent vascular hypothesis for dementia (Ting et al., 2016), with differences in the affected regions. For example, vascular dementia is considered to arise from vascular defects in the white matter (Dichgans and Leys, 2017; Iadecola, 2017). For SCZ, beyond defects in the PFC represented in Figure 1, recent evidence points in the direction of vascular dysfunction in the brain network responsible for treatment of visual stimuli (Lefebvre et al., 2020), possibly resulting in hallucination.

## Conclusion

Projects investigating the immune and vascular components of SCZ in the same protocol are required more than ever to shed light on the pathophysiology of SCZ. This should be approached in more causal studies for the vascular hypothesis to take traction in the SCZ field. A potential avenue would be based on previous work suggesting microvascular damages are coming from hypoxia induced factor 1 after lack of oxygenation during prenatal or early postnatal development (Schmidt-Kastner et al., 2012). This could mean inducing the conditional production of hypoxia induced factor 1 in a double hit protocol to potentially reproduce SCZ-like behavior, thus providing an effective model to the field. The models could then be investigated using 2-photon microscopy to measure blood velocity and glial interactions with the vasculature (Letourneur et al., 2014). Another way would be to directly induce hypoxia in animal models, as done for other pediatric conditions (Johnson et al., 2018; Kiernan et al., 2019) and see if this can reproduce a similar outcome as seen in SCZ patients. In both models, investigating the vascular and the immune dynamic could provide a new understanding leading to novel therapeutic approaches for SCZ.

## Author Contributions

MC was responsible for planning and managing the review, writing of the introduction, discussion, and schizophrenia section while taking care of the overall revision and formatting of the manuscript, and is also the creator of the figure included in the manuscript. JG was in charge of writing the neurovascular unit section and contributing to the neurovascular coupling of the manuscript and on the literature search included in the figure creation. J-PL was responsible for writing the neurovascular coupling section and creating the table. MD and M-ÈT were in charge of revising the manuscript and contributed to the theoretical and writing part of the manuscript while MD contributed significantly to the organization and design of the manuscript. All authors contributed to the article and approved the submitted version.

## Conflict of Interest

The authors declare that the research was conducted in the absence of any commercial or financial relationships that could be construed as a potential conflict of interest.
